# Cargo self-assembly rescues affinity of cell-penetrating peptides to lipid membranes

**DOI:** 10.1038/srep43963

**Published:** 2017-03-06

**Authors:** Andreas Weinberger, Vivien Walter, Sarah R. MacEwan, Tatiana Schmatko, Pierre Muller, André P. Schroder, Ashutosh Chilkoti, Carlos M. Marques

**Affiliations:** 1 Université de Strasbourg, CNRS, ICS UPR 22, F-67000 Strasbourg, France; 2 Duke University, Department of Biomedical Engineering, Durham, North Carolina, USA

## Abstract

Although cationic cell-penetrating peptides (CPPs) are able to bind to cell membranes, thus promoting cell internalization by active pathways, attachment of cargo molecules to CPPs invariably reduces their cellular uptake. We show here that CPP binding to lipid bilayers, a simple model of the cell membrane, can be recovered by designing cargo molecules that self-assemble into spherical micelles and increase the local interfacial density of CPP on the surface of the cargo. Experiments performed on model giant unilamellar vesicles under a confocal laser scanning microscope show that a family of thermally responsive elastin-like polypeptides that exhibit temperature-triggered micellization can promote temperature triggered attachment of the micelles to membranes, thus rescuing by self-assembly the cargo-induced loss of the CPP affinity to bio-membranes.

Cationic cell-penetrating peptides (CPPs) such as oligoarginine (Arg_*x*_) or TAT are non-specific enhancers of translocation across the cell membrane. Their efficiency in crossing cell membranes, independent of cell type^1^, makes CPPs one of the most valuable tools for cellular delivery of cargo molecules. Although the mechanisms of CPP-assisted translocation are not fully understood, it has been suggested that these short arginine-rich peptides facilitate cellular uptake of their associated cargo by active endocytic pathways independent of membrane receptors^2^. CPPs potentially offer two important advantages over receptor mediated endocytosis for delivery of cargo to cells. First, these cationic oligopeptides do not rely on specific features of cellular physiology, such as heterogeneous receptor expression and are thus applicable to diverse cell types. Second, the action of CPPs can be modulated by a variety of external triggers such as the local arginine concentration, pH and temperature^3^.

Interaction with the plasma membrane is the first key step in CPP-assisted translocation. Attractive interactions between CPPs and lipid membranes have been observed for a wide spectrum of simple biomimetic models of the plasma membrane assembled from zwitterionic phopholipids such as DOPC or DOPC/DOPE mixtures, or from mixtures of zwitterionic and anionic phospholipids and cholesterol^4^. Although the affinity of CPPs for membranes appears to hold in a wide range of membrane compositions, membrane permeability to CPPs is only displayed for membranes composed of lipid mixtures with molar fractions of negatively charged lipids that are significantly above typical cell compositions^4^,^5^. This suggests that translocation across the plasma membrane of cells occurs in a two-stage process, where the CPP first binds to the membrane surface and is then translocated across the cell membrane by endocytosis or other energy-driven pathways. Attachment of cargo molecules to CPPs, however, can perturb this process^3^,^6^,^7^. Large macromolecular cargo–peptides, proteins, DNA and polymers–can compromise the attraction of the CPP-cargo to the membrane, either due to size-exclusion or electrostatic effects^1^,^2^,^8^.

Because the ultimate value of CPPs is to transport cargo across the cell membrane, it is critical to understand the molecular details of the interactions of CPP-cargo constructs with plasma membranes to enable the rational design of intracellular delivery systems using CPPs. We have recently developed a family of stimulus responsive diblock copolymers with blocks composed of elastin-like polypeptides (ELP_BC_) with a functional CPP head^9^. Here the ELP_BC_ are the cargo attached to the chosen CPP. These amphiphilic ELP_BC_ are an ideal model system to examine molecular details of translocation of CPPs into cells because: 1) they are recombinantly synthesized from a synthetic gene in *Escherichia coli*, so that their sequence and chain length are precisely specified at their gene level, yielding monodisperse polymers with defined sequence and stereochemistry; 2) different CPPs can be appended to the hydrophilic terminus of the ELP_BC_ at the gene level; 3) these polymers are designed in a diblock architecture with two ELP blocks of different hydrophobicity, and their block composition and length can be tuned to yield ELP_BC_ that undergo micellization at a defined temperature yielding monodisperse micelles with a precisely defined number of polymer chains per micelle^10^. This third feature is of great utility in studying how the cargo architecture controls cell penetration, as self-assembly can be triggered in a relatively narrow temperature range. It allows also to hold constant all other experimental parameters, while morphing the cargo from a unimer polymer chain in a random coil conformation that presents a single CPP moiety at one terminus of the polymer to that of a self-assembled nanoscale object, a spherical micelle that presents multiple copies of the same CPP on its corona. Control of the sequence and chain length of the cargo, the type of CPP and the architecture of the cargo (single unimer polymer chain versus micelle) allows us to examine the effect of cargo properties. We have shown *in vitro* that the self-assembled–micellar state–of ELP_BC_ presenting diverse CPPs can successfully be internalized by several types of cancer cells, while the unassembled state of the ELP_BC_ shows much lower cellular uptake^9^,^11^. This study revealed that translocation of macromolecular–ELP–cargo is rescued by its self-assembly into micelles. However, the complexity of cells precludes a mechanistic understanding of the process by which translocation of the ELP cargo is rescued by its self-assembly into micelles.

In this paper, we study how self-assembly of ELP_BC_ functionalized with arginine-rich CPPs influences their interaction with Giant Unilamellar Vesicles (GUVs) composed either of the zwitterionic lipid DOPC or of the anionic ternary mixture of DOPC/DOPE/DOPG in molar proportions of 65/25/10. We first investigate interactions and the penetration potential of the CPP-functionalized ELP_BC_ with DOPC GUVs in the unimeric state of the ELP_BC_ and compare these results with observations obtained from incubation of vesicles with ELP_BC_ in their micellar state. We show that attachment of cargo to CPPs reduces the well-known affinity of CPP towards neutral lipid membranes, which can be rescued by cargo self-assembly. Finally, we prove that ELP_BC_ containing a high number of arginine units accumulate in their micellar state on the lipid membrane, but that there is no translocation of the cargo, even in the micellar state, into the GUVs. Results obtained by studying the interactions of ELP_BC_ with the anionic ternary mixture DOPC/DOPE/DOPG, mimicking mammalian cell charge and head lipid content follow the same trend and confirm this picture. By inference, this suggests that micelles that present CPPs on their corona are efficiently taken up by the cell, simply because they accumulate at a high local density at the cell membrane which greatly enhances their uptake by active endocytic mechanisms, rather than by direct penetration. This study also explains the mechanism of how cell penetration of CPPs with attached cargo can be rescued by presenting the CPP at very high local density on a self-assembled nanoparticle, and provides a strategy for the rational design of CPP based intracellular delivery systems.

## Results

We used Giant Unilamellar Vesicles (GUVs) composed of DOPC, a zwitterionic lipid, as a model system to investigate lipid-ELP_BC_ interactions. In a typical experiment, the GUVs were incubated in a PBS solution containing 20 *μ*M of ELP_BC_ (See Methods for ELP_BC_ synthesis and Table 1 for physical properties). Interaction behaviour induced by the different CPP functionalities was monitored by confocal microscopy. A small fraction of ELP_BC_, typically between 0.5–1%, was labeled with a fluorophore attached to the extremity of the hydrophobic block. Since the critical micellization temperature (CMT) for our diblock copolymers is in the range of 31–34 °C, we performed experiments in the unimer state at 25 °C and in the micellar state above the CMT.

**Figure 1:**
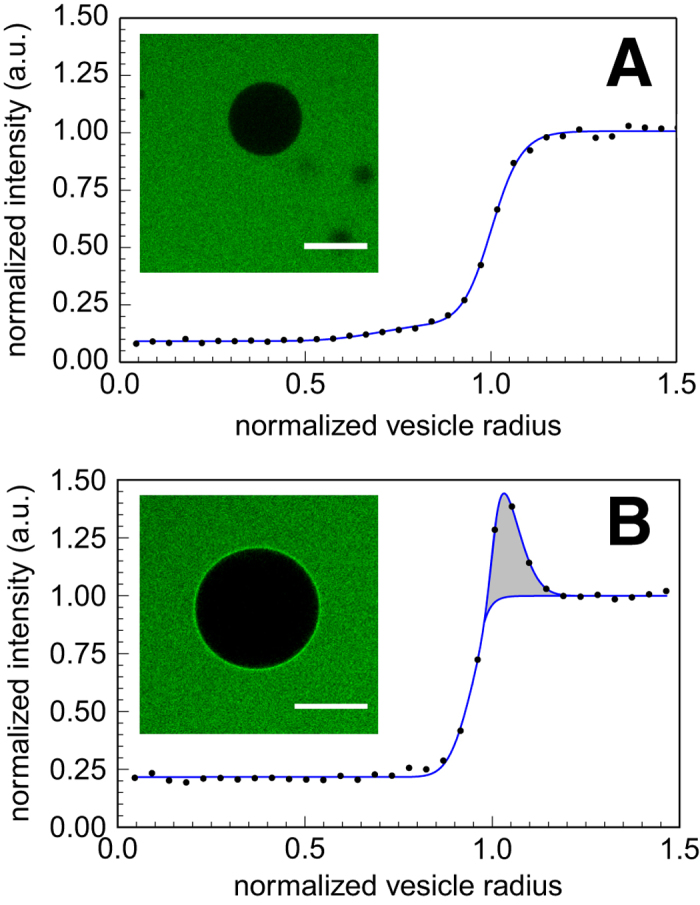
Typical confocal image and intensity radial profiles. Evaluation of the profiles and calculation of the relevant derived quantities are described in the Methods section. (**A**) Radial profile obtained for GUVs dispersed in PBS buffer solution containing functionalized ELP_BC_ unimers at 25 °C. Fluorescence intensity is normalized by the bulk intensity. Vesicle radius (R) is taken at the inflection point of the sigmoidal curve. (**B**) Radial profile across a vesicle after one hour incubation with Arg8- or TAT-functionalized ELP_BC_ in the micellar state above their CMT. The gray area below the peak is proportional to the number per unit surface of ELP_BC_ accumulated on the membrane. Scale bars: 20 *μ*m.

The interactions between ELP_BC_ and lipid membranes were monitored by quantitative analysis of fluorescent intensities in the images acquired by confocal fluorescence microscopy. The radial intensity profile of vesicles was recorded at the equatorial plane. An example of normalized intensity profiles for non-interacting (unimer) ELP_BC_ is shown in Fig. 1A. We first discuss changes in affinity due to attachment of cargo to the CPPs and then study the effect of cargo self-assembly on CPP-membrane interactions.

### Reduction of affinity due to cargo attachment to CPP sequences

ELP_BC_ functionalized with a CPP sequence can be thought of as macromolecular cargo appended to a cell penetrating functionality. The CPP sequences that we used are known to interact favourably with zwitterionic bilayers. For instance, the protein transduction domain TAT accumulates on membranes of GUVs composed from lipids bearing neutral phosphorylcholine head groups even for bulk concentrations as small as 2 *μ*M^4^.

In our experiments, upon incubation of non-functionalized, TAT-, Arg5- or Arg_8_-functionalized ELP_BC_ with DOPC-GUVs in the unimer state at 25 °C, we do not observe any accumulation of ELP_BC_ on the membrane, even for concentrations as high as 20 *μ*M, as shown in the top panel of Fig. 1. Attachment of a polymer cargo to the TAT peptide sequence reduces the known affinity^4^ of CPPs with respect to the membrane, as expected from fundamental arguments of entropy reduction of polymers near interfaces^12^. Indeed, the number of conformations available for a polymer in the bulk solution is larger than those near an impenetrable wall, leading to a reduction of entropy when a chain is brought to the vicinity of the membrane. The associated free energy cost, of the order of a few k_B_T 13, where k_B_ is the Boltzmann constant and T the absolute temperature, can thus cancel out the effective affinity between the phospholipid membrane and the cargo-bearing CPP.

Despite their affinity for zwitterionic membranes, CPPs like TAT are not able to penetrate the bilayers in the absence of a significant amount of charged lipid or phosphoethanolamine head groups^4^. This is consistent with the results of our experiments with TAT and Arg-functionalized ELP_BC_, as we did not observe any permeability of the membranes by CPP-functionalized ELP_BC_. In fact, the residual intensity inside the GUVs does not evolve over time and always exhibits negligible values even when the vesicles are exposed over 24 hours to the unimer ELP_BC_ solution (see Supplementary Table S2 and Supplementary Fig. S1).

### Self assembly rescues the loss of affinity caused by cargo attachment

All of the diblock ELPs included in this study self-assemble into micelles when the solution is heated above their CMT, see Table 1. The non-functionalized ELP_BC_ at this temperature, despite its self-assembly into micelles, does not show any accumulation on the membrane, similar to the observations with unimers of functionalized and non-functionalized ELP_BC_. We next examined the interactions of the micellar assemblies of CPP-functionalized ELP_BC_ with the zwitterionic membranes of DOPC, by evaluating radial intensity profiles of confocal images of GUVs exposed to ELP_BC_ solutions above their CMT.

TAT-functionalized ELP_BC_ self-assemble into micelles and accumulate at the membrane surface, as shown in Fig. 1B. Self-assembly is therefore able to offset cargo-induced reduction of affinity. Several reasons can be invoked to explain such an increase in affinity. Most notably, the micellar aggregate of *p* diblock copolymers has a much lower translational entropy than the corresponding *p* unimers. Therefore, attachment of one micelle implies loosing the entropy corresponding to one degree of freedom, while attachment of *p* unimers would imply loosing a much larger entropy corresponding to *p* degrees of freedom. This is similar to adsorption phenomena of high molecular weight polymers that can adsorb strongly at interfaces even with modest (less than k_B_T) energy of interaction per monomer^12^. Also, the self-assembly of diblock copolymers into a single micelle induces conformational changes in the hydrophilic block of the ELP_BC_, leading to a distribution of the end-functional groups that is more favourable for interactions with a wall^14^.

Arg_8_-functionalized ELP_BC_ display behaviour similar to TAT-functionalized ELP_BC_. Indeed, a quantitative analysis of the fluorescence intensity profiles, explained in the Methods below, revealed similar number coverages for both TAT- and Arg_8_-functionalized ELP_BC_ with N_PTL_, the number of polypeptides per thousand lipids, of the order of 10; more precisely we obtained N_PTL_ = 13 ±  3 for TAT-ELP_BC_ and N_PTL_ = 6 ± 5 for Arg_8_–ELP_BC_. Such values correspond to a surface mass coverage of ~1 mg m^−2^, comparable to the values obtained for moderate strength adsorption of polymers on surfaces^12^.

In contrast, Arg_5_-functionalized ELP_BC_ show no accumulation on the membrane despite their micellar state in solution. Our results thus show that interactions with the membrane can be finely tuned by the arginine content of the CPP that is present at the extremity of the the hydrophilic block of the ELP_BC_.

It is remarkable that despite the increase in affinity for the membrane, the self-assembled micelles of diblock polypeptides do not permeate across the bilayers. Similarly to the case of unimer solutions, the measured relative intensities inside the GUVs are consistently low, even for long periods of incubation (see Supplementary Table S2). The absence of equilibration between inner and outer fluorescence levels gives an upper bound of 10^−4^ *μ*m s^−1^ for the permeability of DOPC bilayers with respect to ELP_BC_, which is several orders of magnitude lower, for instance, than the permeability of DOPC to AF488 at 1.7 10^−2^ *μ*m s^−1^ 15.

We qualitatively observed that the precise kinetics of attachment of ELP_BC_ to the membrane depends on the exact temperature in the observation chamber, but did not precisely study how faster attachment occurs with increased temperature. Despite such effects, attachment always leads to a polypeptide corona that can be detached from the membrane if the temperature is lowered below the CMT. This shows that the binding mechanisms in our case are a result of a chemical equilibrium between the adsorbed layer and the solution.

### Micellar vs. unimer adsorption on the membrane

**Figure 2:**
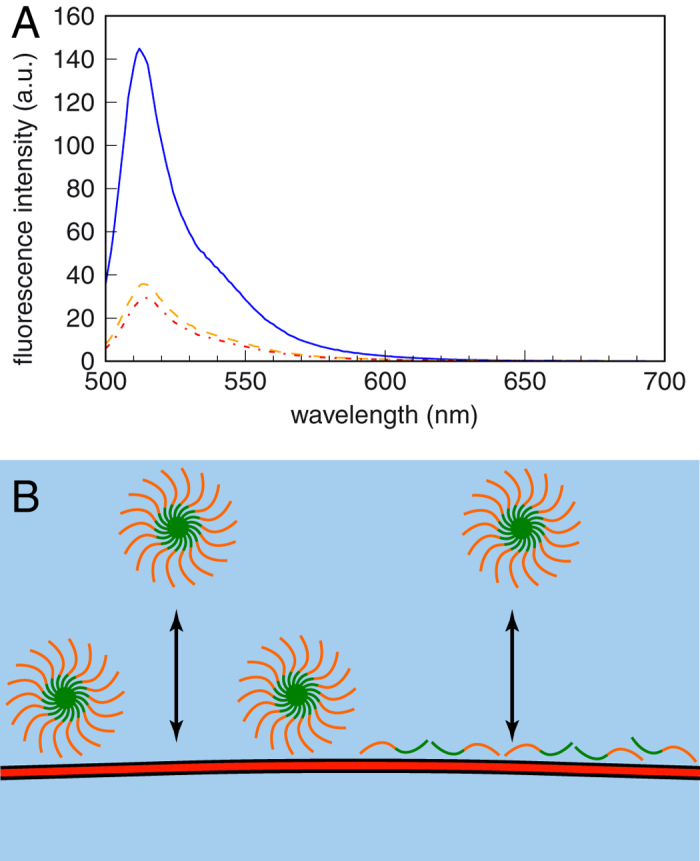
(**A)** Spectrum of a 50% BODIPY-labeled Arg_8_–ELP_BC_ solution in the unimer state at 25 °C (blue solid line) recorded on a spectrofluorimeter. A reduction by self-quenching of around 75% is observed at 40 °C (orange dashed line) and 42 °C (red dashed dotted line). (**B)** Schematic illustration of two possible scenarios for ELP_BC_ adsorption on the phospholipid bilayer, either as micelles or in the unimer state. The arrows emphasize that the attachment process is ruled by the chemical equilibrium between the surface and the bulk^16^.

Polypeptide coverage amounts on the order of 1 mg m^−2^ can be rationalized equally by the attachment of micelles or unimer polypeptides to the membrane, as displayed in Fig. 2B. In order to discriminate between these two possible scenarios, we incorporated BODIPY–a fluorophore that self-quenches significantly at high local concentration–into the ELP_BC_ as shown in Fig. 2A. Indeed, a significant reduction of fluorescence intensity is observed in solution upon assembly into micelles for samples containing 54% BODIPY-labeled ELP_BC_. Note that for samples with fractions of BODIPY-labeled ELP_BC_ below 10%, no quenching is observed.

**Figure 3:**
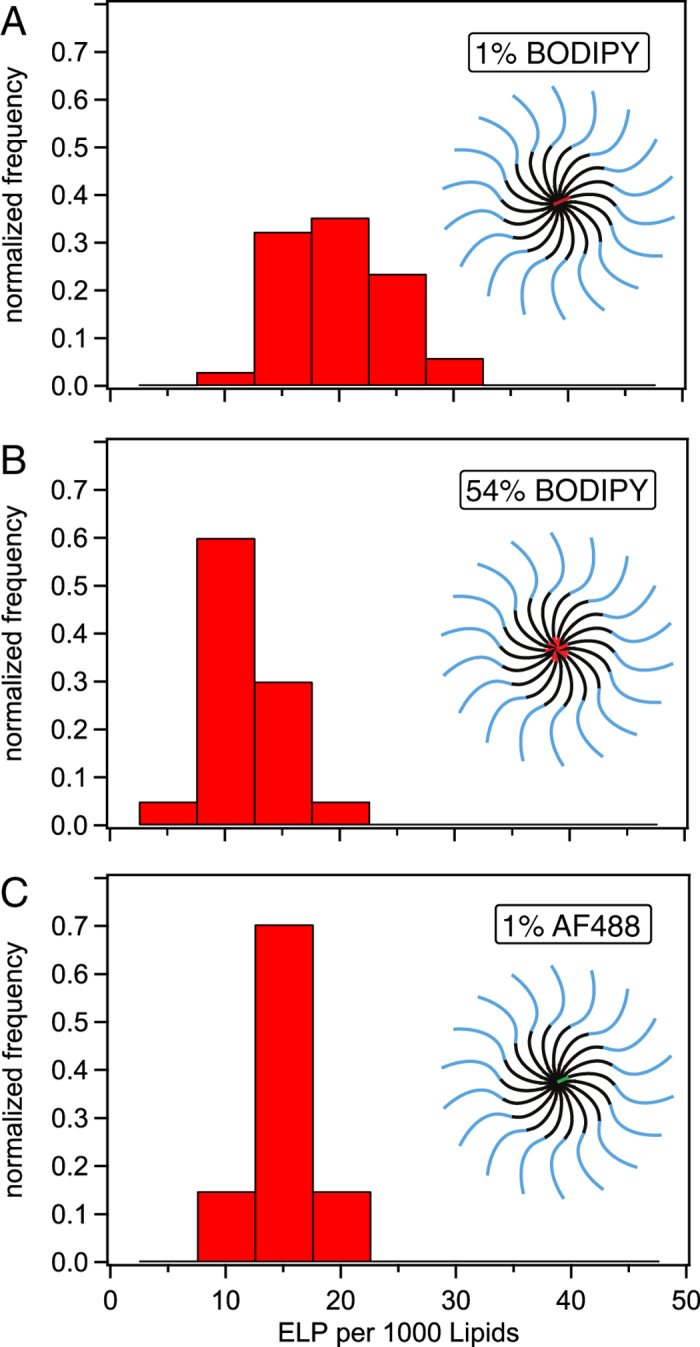
Distribution of N_PTL_ values for samples containing different mixtures of labelled and unlabelled TAT-ELP_BC_. (**A**) with 1% of BODIPY-labeled TAT-ELP_BC_ (**B**) with 54% of BODIPY-labeled TAT-ELP_BC_ and (**C**) with 1% of AF488-labeled TAT-ELP_BC_. Histograms of normalized frequency (the values of the histogram bars add up to one) in (**A**–**C**) were computed from respectively 34, 40 and 27 vesicle profiles. Experiments were performed at 35 °C.

We performed and analyzed experiments under conditions similar to those described above for the AF488. Three different types of ELP_BC_ solutions were prepared: (A) TAT- ELP_BC_ with 1% of BODIPY-labeled TAT-ELP_BC_, (B) TAT-ELP_BC_ with 54% of BODIPY-labeled TAT-ELP_BC_ and (C) TAT-ELP_BC_ with 1% of AF488-labeled TAT- ELP_BC_. Figure 3 shows histograms for the values of surface peptide coverage expressed as N_PTL_, the number of peptides per 1000 lipids–calculation described in the Methods section–obtained for the three types of samples described above. The average number of adsorbed ELP_BC_ per 1000 lipids does not display any significative difference between the three measurement conditions, with an average close to 13 ± 5, corresponding to a mass coverage of ~1.4 ± 0.5 mg m^−2^.

Comparison between the first two histograms in Fig. 3 shows that TAT-functionalized ELP_BC_ adsorb in their micellar form. Indeed, if breakage of the micelles occurred, samples with 54% BODIPY-labeled ELP_BC_ (N_PTL_ = 9 ± 3) should display significantly larger apparent values for N_PTL_ than the sample with 1% of BODIPY-labeled TAT-ELP_BC_ (N_PTL_ = 17 ± 5) due to dequenching at the membrane surface. Note also that samples with 1% of AF488-labeled TAT-ELP_BC_ (N_PTL_ = 13 ± 2) and samples with 1% of BODIPY-labeled TAT-ELP_BC_ (N_PTL_ = 17 ± 5) give comparable results showing that the method is independent of the flurophore.

It is interesting to note that at the polypeptide bulk concentrations (20 *μ*M) where our experiments were performed the surface is not completely saturated with micelles. Indeed, the measured adsorption of micelles corresponds to about half of full coverage (43% ± 16% of occupied projected area) of the membrane surface by micelles, as calculated from an aggregation number of 89 and a radius of 25.9 nm for the TAT-ELP_BC_ micelle. For comparison, if one would cover the surface with a triangular network of a dense packing of monodisperse hard spheres, a coverage of ~90% would be reached.

### DOPC/DOPE/DOPG vesicles

**Figure 4:**
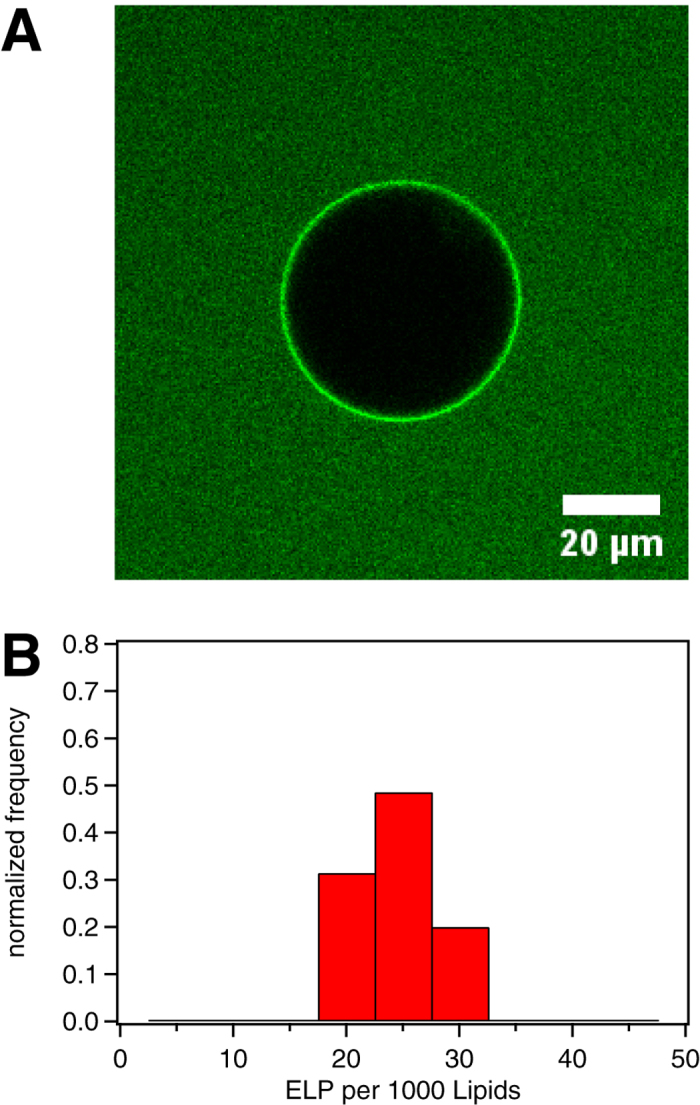
(**A**) Typical image from confocal microscopy of a GUV from a ternary mixture of DOPC/DOPE/DOPG at molar fractions of 65/25/10 exposed to a TAT-functionalized ELP_BC_ above its micellization temperature. (**B**) The corresponding histogram of measured N_PTL_ values from 35 GUVs. Here all experiments were performed at 35 °C.

It has been shown that zwitterionic bilayers enriched with anionic lipids promote the adsorption of TAT without any attached cargo on the lipid membrane, but only lead to vesicle penetration when molar fractions of charged lipids reached values much larger than those that are biologically relevant^4^. Penetration is, however, observed when membranes are prepared from mixtures of DOPC and DOPE, a lipid promoting negative leaflet curvature. We prepared GUVs from the ternary lipid mixture DOPC/DOPE/DOPG at molar fractions of 65/25/10, comparable to the typical phospholipid head composition of mammalian cells. For these systems, we observed a behaviour similar to that of TAT-functionalized ELP_BC_ interacting with zwitterionic membranes. At room temperature, exposure of the GUVs to the solution of the TAT-ELP_BC_ in the unimer form did not lead to the accumulation of peptide on the lipid bilayer, despite of the presence of the negative charges associated with the DOPG, thus confirming a very significant reduction of the CPP affinity to the membrane due to the ELP_BC_ cargo. At temperatures above micellation, an accumulation corona similar to that observed for the zwitterionic membranes also built up, albeit with faster kinetics, typically on the order of ten minutes, and with larger peptide coverage values, N_PTL_ = 22 ± 3, see Fig. 4. Also, similarly to zwitterionic membranes, no translocation of micelles across the ternary membrane was observed, thus further supporting that in cells the observed uptake of ELP_BC_ micelles occurs by active pathways such as endocytosis.

## Discussion

By performing experiments with GUVs composed of the zwitterionic lipid DOPC, we monitored the interactions between the lipid bilayer and a class of cargo-bearing CPPs by fluorescence confocal laser scanning microscopy. Although cationic CPPs display an affinity for bilayers of phosphorylcholine lipids, the attachment of a polymeric cargo like ELP_BC_ suppresses membrane binding interactions. This suppression was also observed for the TAT-functionalized ELP_BC_ with the anionic ternary lipid mixture DOPC/DOPE/DOPG with composition 65/25/10, showing that cargo-induced suppression of binding interactions occurs even with membranes bearing 10% anionic lipid heads.

We have shown that binding to the membrane can be recovered by cargo self-assembly into micelles. The resulting structures, which can be simply pictured as soft nanospheres decorated by CPP sequences, adsorb onto the membrane in their micellar form. However, recovery of affinity depends critically on the number of arginine units in the CPP sequence and it requires more than five arginine residues for this system. Significantly, no penetration was observed either for the unimer or the micellar forms independent of their arginine content. These results on DOPC membranes were also confirmed for the interactions between TAT-functionalized ELP_BC_ and ternary lipid mixtures mimicking mammalian cell compositions. Crucially, this sheds light on previous results, wherein it was shown that self-assembly of CPP-functionalized ELP_BC_ increased cellular uptake for several cell lines^9^,^11^. Our results suggest that uptake is controlled by a first step where self-assembled micelles passively bind to the plasma membrane, before they are actively internalized by cellular mechanisms. The increased uptake of ELP_BC_ micelles bearing Arg_8_ and TAT on their corona is simply due to the increase of interfacial concentration of the micelles at the cell membrane in contrast to the same cargo in its unassembled unimer state.

Our work paves the way not only for designing a strategy for the rational design of CPP based intracellular delivery systems, but provides also a platform for comparing delivery efficiency of CPP based systems with conceptually similar vehicles such as those provided by lipid nanoparticles^17^,^18^.

## Methods

### Materials

Phospholipids used in this work are 1,2-dioleoyl-sn-glycero-3-phosphocholine (DOPC), 1,2-dioleoyl-sn-glycero-3-phosphoethanolamine (DOPE) and 1,2-dioleoyl-sn-glycero-3-phospho-(1′-rac-glycerol) (DOPG) (99%, Avanti-lipids, Alabaster, Al). Lipids were purchased in powder form, dissolved in chloroform and stored at −20 °C before use. Chloroform, sucrose, glucose and phosphate-buffered saline tablets (PBS, 0.01 M phosphate buffer, 0.0027 M KCl and 0.137 M NaCl, pH 7.4) were purchased from Sigma Aldrich as analytical grade chemicals. All of the chemicals were used without further purification.

### Synthesis of functionalized ELP_BC_

ELP_BC_ were named by their CPP-functionalization of the C-terminal of the ELP_BC_: Arg_5_–ELP_BC_, Arg_8_–ELP_BC_, TAT-ELP_BC_ (47YGRKKRRQRRR57) and a ELP_BC_ non-functionalized control. Arg_5_–ELP_BC_ and its respective non-functionalized control ELP_BC_ were genetically designed by Recursive Directional Ligation (RDL) as described elsewhere^19^. Arg_8_–ELP_BC_, TAT-ELP_BC_ and their respective control ELP_BC_ were genetically designed by RDL by plasmid reconstruction (PRe-RDL)^20^. All of the ELP_BC_ possess a ‘leader’ sequence (GCGWPG), the Arg_5_–ELP_BC_ was composed by the following peptide sequence (VPGVG)_60_ -(VPGXG)[X = V:G:A,1:7:8]_96_ and the rest of the ELP_BC_ by (VPGVG)_60_ -(VPGAGVPGGG)_30_.

*E. coli* containing the ELP genes were grown 24 hours at 37 °C while shaking in the presence of ampicillin or kanamycin. *E. coli* were collected by centrifugation and cells were lysed by sonication. ELP_BC_ were purified by their thermal properties by inverse transition cycling^21^. For fluorescent labeling AF488 C5-maleimide or BODIPY FL-maleimide was conjugated to a cysteine residue on the N-terminus of the ELP_BC_ in the presence of 3 mM TCEP-HCl and 10 mM NaH_2_PO_4_ at a pH of 7 for 2 hours. Aggregation of ELP_BC_ was induced by addition of NaCl, the labeled ELP_BC_ was collected by centrifugation and the supernatant was discarded. Afterwards the ELP_BC_ were resuspended in PBS buffer solution and remaining free fluorophore was removed with a desalting column. Dialysis was performed against water and the ELP_BC_ were lyophilized. The lyophilized ELP_BC_ were stored at −20 °C and resuspended in PBS solution at a concentration between 16–25 *μ*M before use.

The temperature dependent self-assembly of ELP_BC_ at 25 *μ*M in PBS buffer was monitored with dynamic light scattering (DLS) in the range between 25 °C and 50 °C. The critical micellar temperature and the hydrodynamic radius were determined, see Table 1. The aggregation number was determined by static light scattering (SLS) at 37 °C. Supplementary Fig. S2 shows an example of a recorded temperature dependent DLS. Values of the CMT and the hydrodynamic radius of the micelles are insensitive to conjugation of fluorophore.

### Preparation of GUVs

GUVs were prepared by following the electroformation method of Angelova *et al*.^22^. Briefly, 10 *μ*L of the lipid solution (1 mg ml^−1^ in chloroform) were spread on an indium tin oxide (ITO) coated glass slide. After drying the lipid film under vacuum for 30 min, a chamber was formed with a second ITO slide and Sigillum wax (Vitrex, Copenhagen, Denmark) as sealing agent. This chamber was filled with sucrose solution. The osmolarity of the solutions was measured with an osmometer (Osmomat 030, Gonotec, Berlin, Germany) and adjusted to 280 mOsm kg^−1^. An alternating electric field was applied across the chamber for 3–12 hours at room temperature. The amplitude and the frequency of the field were set to 1 V and 10 Hz. Successful formation was checked by observing the growing chamber by phase contrast microscopy. The obtained GUVs were transferred to an Eppendorf tube, diluted one time with isoosmotic PBS or glucose solution and left undisturbed for 15 min before further use.

### Interactions of ELP_BC_ with GUVs

The ELP_BC_ were dissolved in a 280 mOsm kg^−1^ PBS solution in a concentration range of 16 to 25 *μ*M. The solutions were adjusted such that around 0.5% of the ELP_BC_ were fluorescently labeled with AF488. For a typical experiment three volume parts of the solution containing the ELP_BC_ were mixed with one volume of the previous prepared vesicle solution. Parallel samples were usually prepared and incubated at 25 °C (below CMT) and at 35 °C (above CMT). Samples were incubated for 1.5–2 hours directly under the confocal microscope, using a home made heating cell (Supplementary Fig. S3). Out of focus light was minimized by imaging the GUV at the equator of the vesicle.

The observation directly under the microscope allowed additionally the measurement of the partition coefficient of a single GUV as a function of time. After incubation, image recording of random GUVs within the sample was started and usually finished after a maximum of two hours for the whole experiment.

### Optical microscopy

Vesicle contours were imaged by phase contrast microscopy or differential interference contrast microscopy (DIC) using an inverted TE 2000 microscope (Nikon, Japan) equipped with a 60x WI/1.2NA Plan Apo DIC objective or 40x Ph2/NA 0.60 Plan Fluor objective. Images were recorded with a digital camera (Hamamatsu EM-CCD, Japan) with a pixel depth of sixteen bits. The reflection interference contrast microscopy technique (RICM) was used with a 100x NA 1.4 Plan Apo objective for locating adhered vesicles (Supplementary Fig. S5).

Fluorescent imaging was performed using confocal laser scanning microscopy (CLSM) with a Nikon C1 scanhead. Images were captured using EZ-C1 software (Nikon, version 3.50). The AF488 and BODIPY labeled ELP_BC_ were excited using a argon-ion laser (Melles-Griot) at 488 nm.

### Quantitative measurements of adsorbed amount from image analysis

Quantitative analysis of fluorescent intensities in the confocal images was performed using radial, angularly averaged, intensity profiles extracted with the “radial profile extended plugin” from Philippe Carl from the ImageJ homepage. To ensure that the measured fluorescence intensity of the fluorescently labeled ELP_BC_ behaves linearly with the concentration of the fluorophores, a fluorescence intensity curve was recorded with the confocal microscope at different heights from the glass slides (Supplementary Fig. S4). Furthermore, in order to properly account for possibly different acquisition parameters for different experiments, a calibration method (Supplementary Methods) was developed. For quantification of the adsorbed ELP_BC_ amount, the excess fluorescence above the level of sigmoidal profile for non decorated membranes was evaluated and Γ, the number of ELP_BC_ per unit membrane surface was extracted from


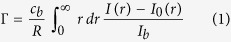


where *I*(*r*) is the radial profile intensity in the presence of excess coronal intensity, *I*_0_(*r*) the intensity profile in absence of corona, *I*_*b*_ = *I*(*r* → ∞) = *I*_0_(*r* → ∞) the bulk intensity, *c*_*b*_ the bulk number concentration of the ELP_BC_ and *R* the radius of the vesicle. Equation 1 assumes equal quantum yields for fluorophores in the bulk and in the vicinity of the membrane, a valid assumption for fluorophores which do not penetrate the membrane, at fluorophore surface concentrations below self-quenching values. For our particular case we convert Γ to the more informative quantity *N*_PTL_, the number of polypeptides adsorbed per thousand lipids by using


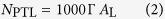


with *A*_L_ the area of a lipid. Given that for DOPC *A*_L_ ≃ 0.7 nm^2^, *N*_PTL_ represents here the number of peptides in a lipid patch of roughly 25 × 25 square nanometers.

## Additional Information

**How to cite this article:** Weinberger, A. *et al*. Cargo self-assembly rescues affinity of cell-penetrating peptides to lipid membranes. *Sci. Rep.* **7**, 43963; doi: 10.1038/srep43963 (2017).

**Publisher's note:** Springer Nature remains neutral with regard to jurisdictional claims in published maps and institutional affiliations.

## Supplementary Material

Supplementary Information

## Figures and Tables

**Table 1:** ELP_BC_ and corresponding physical properties in the unimer (25 °C) and micellar state (35 °C).

ELP construct (hydrophobic/hydrophilic) pentapeptide block ratios	C-terminal functionality	MW (kDa)	CMT °C	R_h_(nm) 25 °C*	R_h_(nm) 35 °C*
ELP_BC_ (60/96)	none	61.8	33	6.8 ± 0.1	25.1 ± 0.9
Arg_5_-ELP_BC_ (60/96)	Arg_5_	62.6	33	6.8 ± 0.2	26.9 ± 0.6
ELP_BC_ (60/60)	none	48.0	34	5.9 ± 0.8	24.0 ± 1.1
Arg_8_-ELP_BC_ (60/60)	Arg_8_	49.3	31	7.5 ± 0.2	23.5 ± 0.3
TAT-ELP_BC_ (60/60)	TAT	49.6	32	6.0 ± 0.7	25.9 ± 1.4

All the samples were also available with an Alexa Fluor 488 fluorophore (AF488) attached to the extremity of the hydrophobic block (N-terminal). The last 3 samples in the table were also prepared with a BODIPY fluorophore instead of AF488. *ELP_BC_ at 20 *μ*M in PBS. Data represents the average of 3 replicates ± standard deviations.
