# Supplementary material for: Cargo self-assembly rescues affinity of cell-penetrating peptides to lipid membranes
Source: Sci Rep. 2017 Mar 6;7:43963. doi: 10.1038/srep43963 (PMC5338251; doi:10.1038/srep43963)
Supplement: Supplementary Information [file srep43963-s1.pdf]

Supplementary Information for: Cargo self-assembly rescues affinity of cell-penetrating peptides to lipid membranes

Andreas Weinberger[1,+], Vivien Walter[1,+], Sarah R. MacEwan[2,3], Tatiana Schmatko[1], Pierre Muller[1], André P. Schröder[1], Ashutosh Chilkoti[2], and Carlos M. Marques[1,*]

[1]Université de Strasbourg, CNRS, ICS UPR 22, F-67000 Strasbourg, France
[2]Duke University, Department of Biomedical Engineering, Durham, North Carolina, United States
[3]Current address: University of Chicago, Institute for Molecular Engineering, Chicago, Illinois, United States
[*]marques@unistra.fr
[+]these authors contributed equally to this work

Supplementary Tables

Table S1. Acronyms used in this paper

AF488	Alexa Fluor 488
Arg_x	Oligoarginine with x arginines
BODIPY	Boron-Dipyrromethene
CMT	Critical Micellization Temperature
CPP	Cell Penetrating Peptide
DIC	Differential Interference Contrast
DOPC	1,2-dioleoyl-sn-glycero-3-phosphocholine
DOPE	1,2-dioleoyl-sn-glycero-3-phosphoethanolamine
DOPG	1,2-dioleoyl-sn-glycero-3-phospho-(1'-rac-glycerol)
ELP	Elastin-like Polypeptide
ELP_{BC}	Elastin-like Polypeptide block copolymer
GUV	Giant Unilamellar Vesicle
N_{PTL}	Number of Polypeptides adsorbed per Thousand Lipids
RICM	Reflexion Interference Contrast Microscopy
PM	Photomultiplier
TAT	Trans-Activator of Transcription

Table S2 summarizes the relative intensities inside the vesicles obtained for incubation with functionalized ELP_{BC} above and below the CMT of the ELP_{BC}. The non-vanishing values are likely to be related both to the mixing process during sample preparation and to light pollution from the glass surfaces when the GUVs are strongly adsorbed, see Figure S5. By tracking a single vesicle during an incubation time of 1 hour after mixing, it could be shown that the internal intensity does not increase with incubation time (Figure S1). Longer incubation time of up to 24 hours did not lead to any further increase of the internal intensity. For experiments performed with ELP_{BC} in their micellar state above the CMT an increased dispersion of the values and a higher initial intensity value could be observed. The higher initial values are likely to be determined during mixing.

Table S2. Relative GUV internal intensity in % for different types of ELP_{BC} incubated with GUVs of DOPC at 25 °C and above CMT. Relative intensity values do not correlate with incubation times that range here from 2 to 24 hours.

ELP-Type	Fluorophore	25 °C		above CMT	
		Mean	SD	Mean	SD
Arg_5	AF488	7.12	2.79	8.86	4.26
Arg_8		9.00	2.16	16.22	8.59
TAT		8.18	1.33	20.53	13.33
No function		5.79	2.37	8.26	3.34
Arg_8	BODIPY	6.93	1.52	19.42	9.89
No function				21.07	5.9

Supplementary Figures

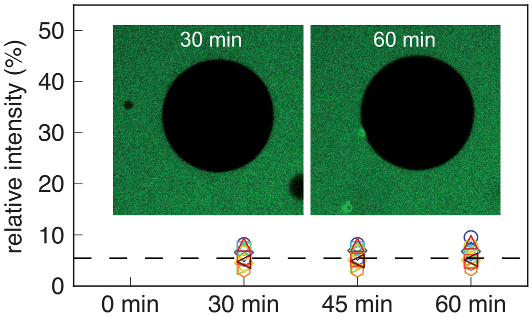

Figure S1. No change of relative internal fluorescence intensities within one hour. After 30 min of stabilization, recording was started. The confocal pictures show one DOPC vesicle surrounded by a PBS Buffer solution containing Arg_5-functionalized ELP_{BC} at 40 °C, similar results are obtained with other functionalizations. No change in relative intensities can be observed. Each symbol stands for one single GUV per sample. Different symbols signify different functionalities and incubation temperatures. Vesicle diameter is 35 μm.

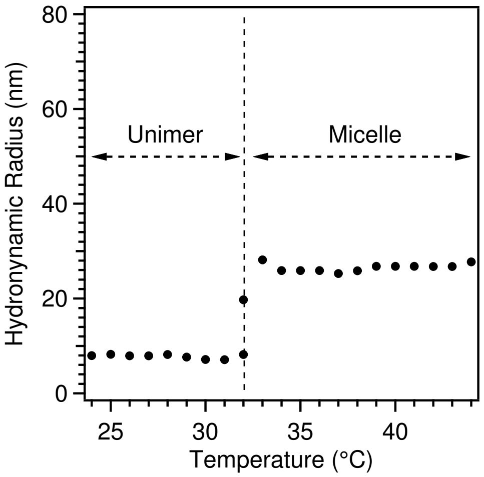

Figure S2. ELP_{BC} form micelles above the CMT as shown by measurement of the hydrodynamic radius by DLS. Soluble unimers of TAT-ELP_{BC} existed with a R_h below 10 nm up to 32 °C. At 32 °C self-assembly into micelles occurred. Micelles with a R_h of approximately 30 nm were formed. At temperatures above 50 °C micrometer size aggregates were formed. Measurements at 25 °C and 37 °C were repeated five times, with measured deviations of about 2%.

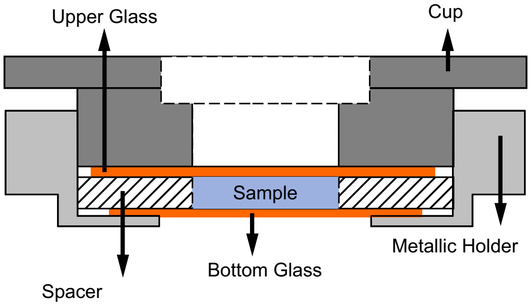

Figure S3. Home-made heating cell for observations and incubation directly under the microscope. The objective under the cell is also temperature-regulated. Due to heat loss, the temperature of the heating bath that warms the metallic holders had to be adjusted for different experiments, to ensure appropriate temperatures in the chamber within the micellar range of the ELP_{BC}.

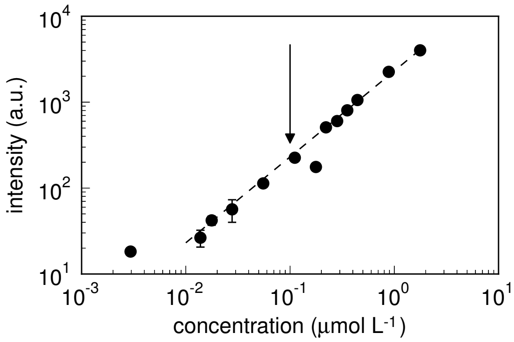

Figure S4. Fluorescence calibration curve for a solution of a control ELP_{BC} labeled with AF488 at room temperature, with the intensity given as a function of fluorophore concentration. The arrow represents the typical working conditions. The values were measured at 20 μm, 50 μm, 80 μm and 130 μm from the glass surface. Points represent the mean value of the four different positions and errorbars the standard deviation.

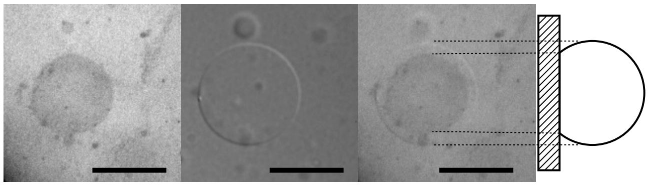

Figure S5. RICM (left) and DIC (center) images of GUVs incubated in TAT and Arg_8 functionalized ELP_{BC} solution above the CMT. Additionally to the appearance of the corona (as described in the main text), GUVs started to adhere on the glass surface of the heating cell, as observed by RICM, indicating attractive interactions between the CPP residues, the membrane and the glass surface. The right image superimposes the RICM and the DIC images. Scale bars 20 μm.

Supplementary Methods: calibration of the confocal images

The proportionality between the local sample concentration of fluorophores and the intensity displayed in the confocal image is a key requirement to extract the adsorbed fluorophore amount per unit surface of the membrane, a quantity in our work better expressed as the number of polypeptides adsorbed per thousand lipids or N_{PTL}. It is easy to realise however that analysis of images collected from the same vesicle for different acquisition parameters, such as the photomultiplier gain or the laser power might not yield the same N_{PTL} values even after the trivial expected corrections, see Fig. S6. Here, we present a method to easily account for these variations and assure the extraction of proper N_{PTL} values from the images.

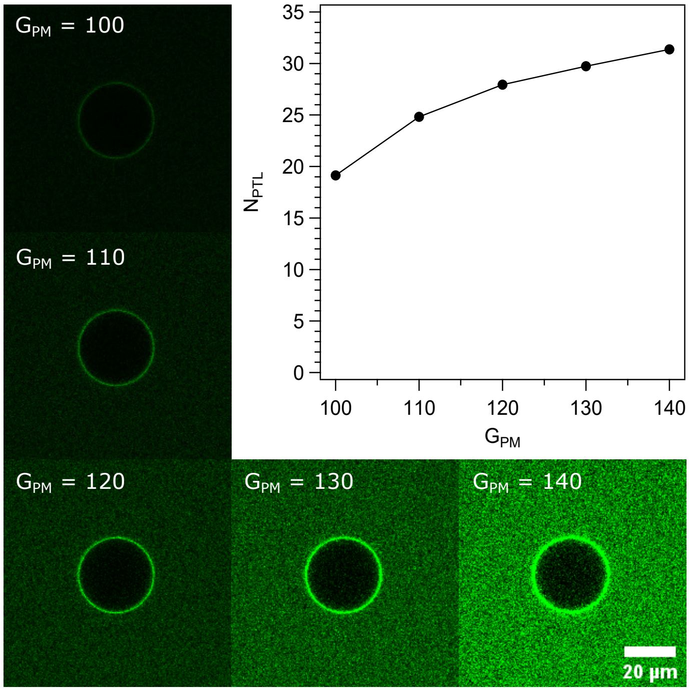

Figure S6. N_{PTL} measured from the raw intensity radial profile of the same vesicle, taken with different photomultiplier gains. Pictures display the PM gain G_{PM} used for acquisition, ranging from 100 to 140. The objective of the calibration procedure is to ensure stable measurement of N_{PTL} for all practical acquisition conditions.

The settings of the confocal microscope

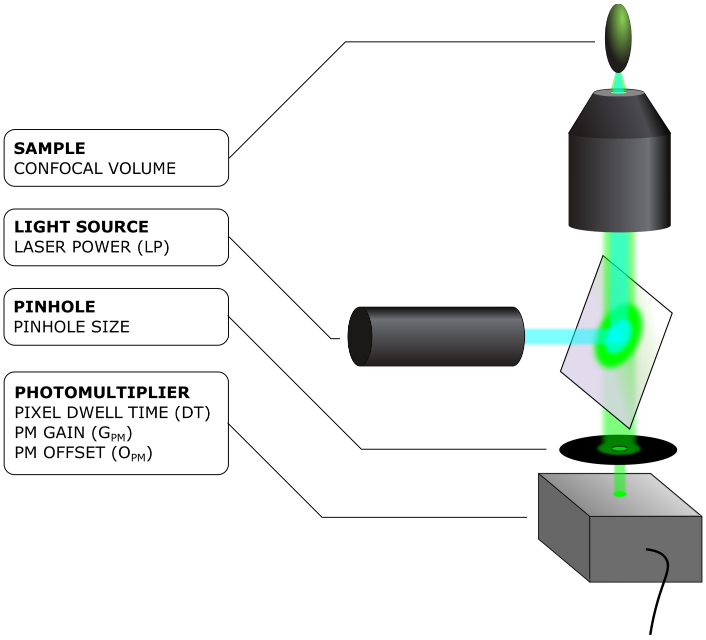

Figure S7. Simplified representation of the elements of the confocal microscope, highlighting the different parameters and settings which have an impact on the measured fluorescence intensity. All experiments are performed with the same objective at a fixed pinhole size.

The different parameters controlling the intensity value $I_P(x,y)$ acquired for each image pixel at the point (x,y) are shown in Fig. S7. Although the photomultiplier (PM) responds linearly to the number of collected photons, albeit with an offset O_{PM},

several spurious factors such as electronic noise contribute also to a background of the signal and need to be accounted for. In general the recorded pixel intensity value I_P is thus related to the relevant intensity I through

$$I_P(x,y) = A(G_{PM}, DT)I(x,y) + B(O_{PM}, DT) \quad \text{(S1)}$$

where $A(G_{PM}, DT)$ is a prefactor that depends on the PM gain G_{PM} and on the dwell time DT. The background factor $B(O_{PM}, DT)$ depends on the PM offset O_{PM} and on DT. The laser power LP changes also the pixel value I_P because the sought intensity I is proportional to LP, which of course does not perturb the calculation of N_{PTL}. Consistently we also found that the factors A and B in equation S1 do not depend on LP.

Calibration

The calibration procedure amounts to determine the factors $A(G_{PM}, DT)$ and $B(O_{PM}, DT)$ of supplementary equation S1. The first part of the calibration was done with a reference non-fluorescent sample (a standard buffer without fluorophores), so that in equation S1 one has $I_P = B(O_{PM}, DT)$. By measuring pixel values I_P while changing the settings of the microscope allows not only to extract the function $B(O_{PM}, DT)$ – see figure S8 – but also to confirm that the background value does not depend on laser power, PM gain or even on pinhole size.

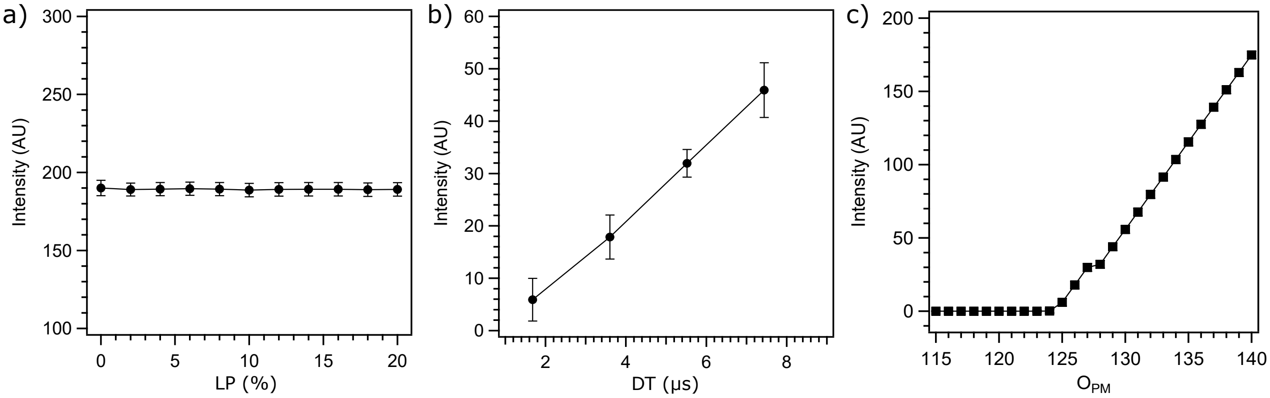

Figure S8. Calibration with a reference non-fluorescent sample, to determine the factor $B(O_{PM}, DT)$ of equation S1. a) The measured intensity I_P is independent of the Laser Power LP. Intensity acquired with O_{PM}=150 and DT=3.6 μs; b) Variation of I_P with the Dwell Time DT. Here O_{PM}=128; c) The importance of the PM offset parameter O_{PM}. Here DT=5.52 μs.

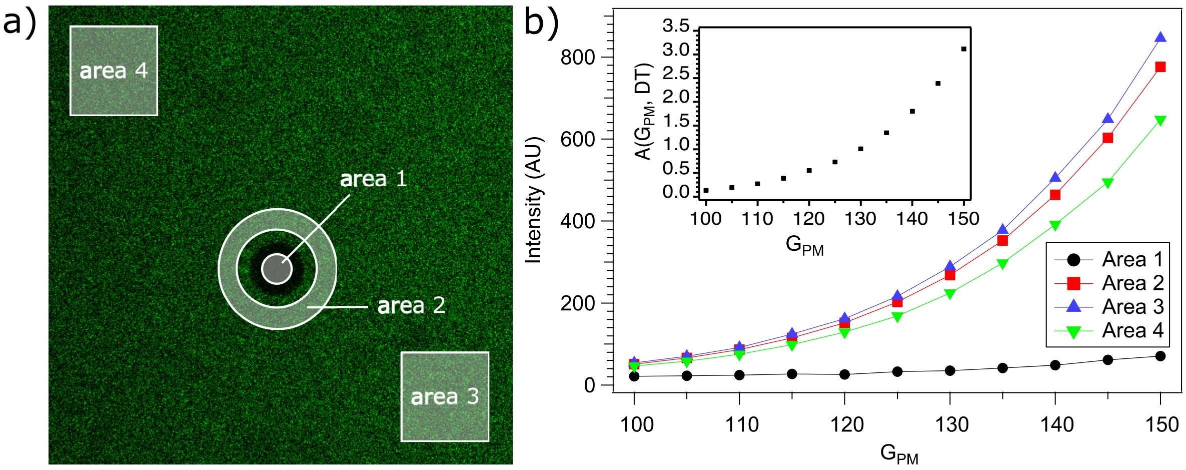

Figure S9. Calibration with a fluorescent sample containing non-fluorescent GUVs, to determine the factor $A(G_{PM}, DT)$ of equation S1. a) The four zones of an image under analysis. b) Intensities of the four zones for different photomultiplier gains G_{PM} at constant DT=5.52 μs and O_{PM}=128. The factor $A(G_{PM}, DT)$ obtained from the calibration is shown in the inset.

The second part of the calibration consists in extracting the factor $A(G_{PM}, DT)$ by analysing the pixel values I_P of a number of different spots (four in our case) of images from a fluorescent solution containing non-fluorescent GUVs while changing

the value of the different acquisition parameters, see figure S9 for an example where the PM gain is changed at constant DT=5.52 μs and O_{PM}=128. Images from this system, where intensities vary due both to inhomogeneous light and fluorophore distributions, conveniently provides for images with a range of intensities and thus allows for acquiring different intensity values with the same image. The calibration consists of using supplementary equation S1 to find the function $A(G_{\mathrm{PM}}, DT)$ that best gives identical "true" I values at the four points from the sample. The inset in figure S9 shows the obtained factor $A(G_{\mathrm{PM}}, DT)$ for our particular case.

Checking the calibration

After calibration, supplementary equation S1 can be routinely used to extract the relevant values of sought intensity I. Also, one can check that derived quantities such as the intensity radial profiles normalized by the intensity of the bulk are independent of the acquisition parameters. An example of such a check is displayed in supplementary Fig. S10 for two different PM gains.

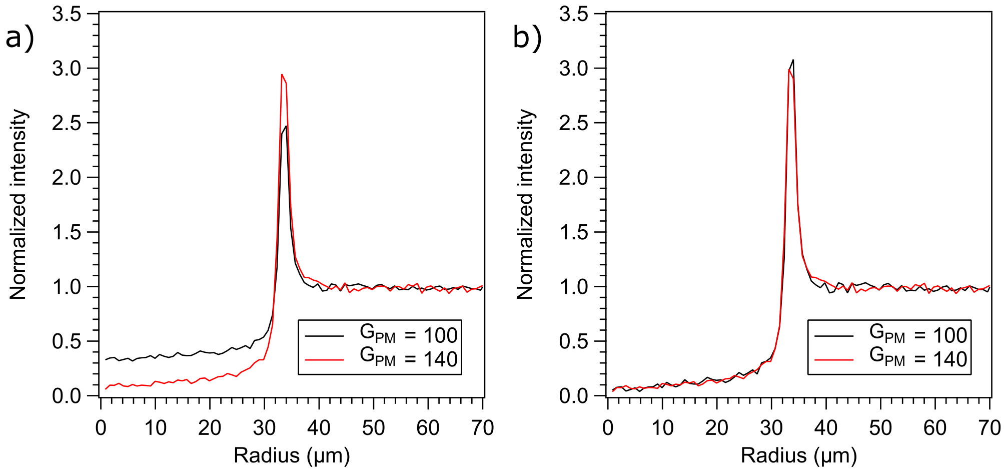

Figure S10. Intensity radial profiles acquired for two different PM gains and normalised with respect to the intensity of the bulk. (a) Raw values of the intensity. (b) Intensities after correction by the calibration equation S1.

Most importantly, the derived quantity N_{PTL} which is of relevance for our study, can now be computed independently of the acquisition parameters. As an example, we compare in supplementary figure S11 the values corresponding to the supplementary figure S6 before and after the calibration procedure.

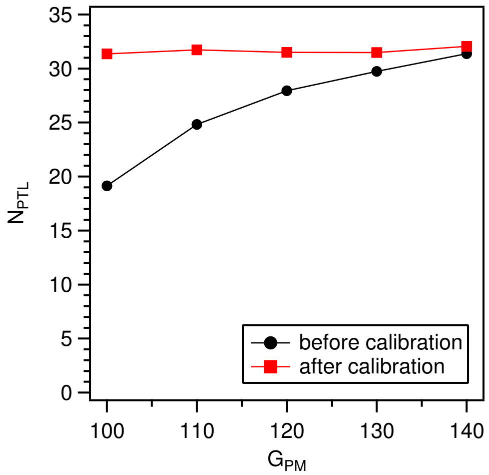

Figure S11. Comparison of the N_{PTL} values corresponding to images in figure S6, before and after the calibration procedure.
